# Methods to collect *Anopheles *mosquitoes and evaluate malaria transmission: A comparative study in two villages in Senegal

**DOI:** 10.1186/1475-2875-10-270

**Published:** 2011-09-19

**Authors:** Mamadou O Ndiath, Catherine Mazenot, Ablaye Gaye, Lassana Konate, Charles Bouganali, Ousmane Faye, Cheikh Sokhna, Jean-Francois Trape

**Affiliations:** 1 Institut de Recherche pour le Développement, UMR 198 URMITE Campus international de Hann, IRD BP 1386 CP 18524 Dakar, Sénégal; 2 Université Cheikh Anta Diop de Dakar, Département de Biologie Animale, BP 5005 Dakar, Sénégal

## Abstract

**Background:**

Various methods have been studied as replacement of human landing catches (HLC) for mosquito sampling in entomological studies on malaria transmission. Conflicting results have been obtained in comparing relative efficiency of alternative methods, according to the area, the species present and their density. The aim of this study was to compare the number and characteristics of mosquitoes sampled in two areas of Senegal by three different methods: HLC, light traps adjacent to an occupied bed net (LT/N), pyrethrum spray catches (PSC).

**Methods:**

Collections were performed in two villages: Dielmo (Soudan savanna) and Bandafassi (Soudan Guinean savanna), two or three nights per month for a 4-5 months period during the maximal transmission season in 2001-2002. Species were identified and *Plasmodium *infection determined by ELISA. The specific composition, circumsporozoite protein rate and entomological inoculation rate were calculated.

**Results:**

The diversity of mosquito species captured was maximal with LT/N, minimal with PSC. The mean number of anopheles captures each night was significantly different according to the method used and the species. PSC displayed a significantly lower anopheles density. HLC was the most efficient sampling method when *Anopheles gambiae *was the main vector (in Bandafassi); LT/N when it was *Anopheles funestus *(in Dielmo). A significant correlation was found between HLC and LT/M but correlation parameters were different according to the species. Circumsporozoite protein rates were not significantly different between methods or species. The entomological inoculation rate varied along with vector density and thus with methods and species.

**Conclusions:**

The choice of sampling method influenced entomological data recorded. Therefore, the sampling technique has to be chosen according to the vector studied and the aim of the study. Only HLC must be considered as the reference method, but in some conditions LT/N can be used as an alternative method.

## Background

In order to measure malaria transmission, a good knowledge about its vectors is required. To achieve this goal, entomological studies with *Anopheles *collection are essential [1]. The choice of the method depends on the objectives of the study, the environment and the available means [2].

Human landing catch (HLC) is the most frequently used and considered as the reference method. It allows sampling mosquitoes that are aggressive against human, either endophagous or exophagous. It is the most reliable measure of human-vector contact for evaluating malaria transmission. On the other hand, it raises the ethical question of potential risk for collectors that are submitted to mosquito bite susceptible to transmit various pathogens. Results depend on collectors skills and on the attraction he/she exerts on mosquitoes. Several alternative sampling methods have been developed: use of various traps, light traps (CDC miniature light trap) [3] with or without a person sleeping under a net, CO_2 _[4] or odour-baited traps (OBET) [5], exposure free bed-net traps (Mbita) [6] and indoor resting catches by aspiration or spraying. Because they select only a fraction of the global anopheles population, each method is subjected to bias and shortcomings and, therefore, influences the results [7]. Specimens sampled by pyrethrum spray catches (PSC) are mostly fed females resting indoor in the morning. The use of traps, as the CDC Light Trap, associated with a person sleeping under a net (LT/N) should theoretically allows sampling the anthropophilic and endophagous specimens that are searched. However, the presence of light attracts other species that are not anthropophilic. Collections with LT/N should allow a good standardization.

Malaria epidemiologic studies are currently performed in Dielmo and Bandafassi, two Senegalese villages. In these two sites, different vector species are present [8,9]. The aim of this study was to evaluate relative efficacy of three collection methods: Light traps associated with a person sleeping under a net (LT/N), pyrethrum spray catches (PSC) and Human Landing Catches (HLC).

## Methods

### Study area

The village of Dielmo (13°45'N, 16°25'W) is situated 280 km south-east from Dakar, near the Gambian border. Rainfalls (around 630 mm/year) occur between June and October. A small permanent stream situated near the village constitutes a site for *Anopheles *larval growth. Malaria is holoendemic and transmission occurs all the year round [10]. The village of Bandafassi (12°33'N, 12°17'W) is situated 730 km south-east from Dakar. Rainfalls (1,500 mm/year) occur between May and November. Malaria is hyperendemic with long seasonal transmission [11].

### Sampling methods

Collections were performed three times per month from October 2001 to January 2002 in Dielmo and twice monthly from July to October 2002 in Bandafassi. Night catches were performed simultaneously indoor between 7 PM and 7 AM by HLC and LT/N methods. HLC were performed by two trained collectors (adult male volunteers) working alternatively for one hour and resting for one hour. Village nurses provided medical supervision of collectors. LT/N catches were performed using a CDC mini light trap [3] placed adjacently and above an occupied bed net. PSCs were performed at 7 AM by spraying Deltamethrin (Yotox^®^) for 30-45 seconds in the room. After 10 minutes, dead and immobilized mosquitoes were collected. Two sites per villages were randomly selected. In each site, three rooms were randomly chosen within a 15-m distance. Each night, a different sampling method was tested in each room.

### Mosquito analysis

After collection, specimens were brought back to the field laboratory and *Anopheles *morphologically identified according to Gillies and DeMeillon keys [12]. Females were counted and stored for further analysis. The expression of circumsporozoite protein (CSP) was assessed by ELISA in the laboratory in Dakar [13].

### Data analyses

For each method, the number of species, human biting rate (HBR) or *Anopheles *density (number of *Anopheles *per person and per night) and CSP rate were calculated. The entomological inoculation rate (EIR) was defined as the number of *Anopheles *person and per night multiplied by the CSP rate and expressed in number of infected bite per person per night. The mean number of mosquitoes collected per night was compared by ANOVA with following factors (village, method and species) after log+1 normalization (Shapiro-Wilk test) with post hoc Bonferroni test. CSP rates were compared using Pearson or Fisher Chi^2^. The correlation between LT/N and HLC was studied with Spearman test.

### Ethical approval

Free and informed consent was obtained from collectors performing HLC and LT/N. Permission was sought from inhabitants to perform collections in their rooms. Community consent had been obtained beforehand in both villages. This study was approved by the Ethical National Comity of Senegal.

## Results

### Anopheles density and diversity

In Dielmo, LT/N was the method that allowed collecting the highest variety and quantity of *Anopheles *(1,164 specimens belonging to five different species). HLC method gave lower results with 897 specimens belonging to three different species. Only 439 specimens belonging to two different species were collected by PSC.

In Bandafassi, similar results were obtained with nine different species collected by LT/N, 6 by HLC. However, the number of specimens collected was higher with HLC (1841 vs. 1061 with LT/N). For variety (4 different species) as well as for quantity (444) PSC was the method that displayed the lowest result (Table 1).

**Table 1:** Number of *Anopheles *collected in Dielmo and in Bandafassi according to species and method used (HLC: human landing catches, PSC: pyrethrum spray catches, LT/N Light trap associated with a person sleeping under a net)

	DIELMO			BANDAFASSI		
	
	HLC	PSC	LT/N	HLC	PSC	LT/N
*An. gambiae s.l.*	219	164	169	1555	391	953
*An. funestus*	676	275	983	56	33	54
*An. nili*	-	-	-	223	14	30
*An. ziemanni*	-	-	7	1	-	11
*An. coustani*	-	-	-	3	-	2
*An. domicola*	-	-	-	3	-	3
*An. pharoensis*	2	-	1	-	-	5
*An. rufipes*	-	-	4	-	6	2
*An. paludis*	-	-	-	-	-	1

*Total*	897	439	1164	1841	444	1061

**Figure 1:**
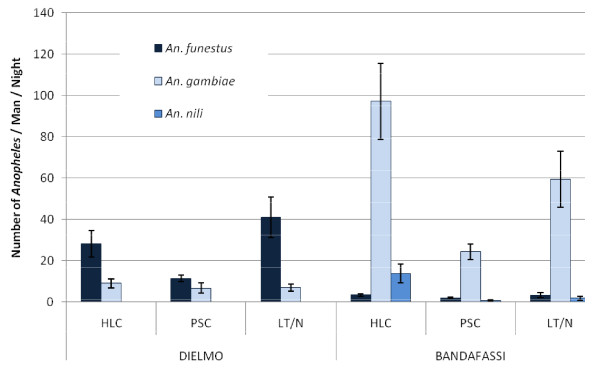
**Mean ± s.e.m. number of *Anopheles *collected per man and per night according to the method (HLC: human landing catches, PSC: pyrethrum spray catches, LT/N Light trap associated with a person sleeping under a net), the village (Dielmo and Bandafassi) and the *Anopheles *species (*An. funestus, An. gambiae *and *An. nili*)**.

The mean number of *Anopheles *collected per person and per night is represented on Figure 1. Analysis demonstrated a significant difference according to species (F = 32.95, p < 0.0001). In Dielmo, significantly more *Anopheles funestus *than *Anopheles gambiae *were collected (p < 0.0001, Bonferroni test). In Bandafassi, *An. gambiae *was the most encountered species (p < 0.0001, Bonferroni test). A significant difference was demonstrated between methods (F = 14.19, p < 0.0001) with HLC being significantly more efficient than PSC (p = 0.005, Bonferroni test). No significant difference was observed between villages (F = 0.12, p = 0.7); on the other hand, a strong interaction was identified between village and species (F = 139.72, p < 0.0001). For *An. gambiae *the relative efficiency of LT/N using HLC as reference was 0.6. For *An. funestus *it was 1.4.

### CSP rates

The number of CSP positive mosquitoes and CSP rates are presented in Table 2. Infection rates were similar for all species when analysed in Dielmo (Fisher exact p = 0.5) or in Bandafassi (Fisher exact p = 0.28). No significant difference could be identified between methods in Dielmo and in Bandafassi (Pearson Chi^2 ^respectively 0.7, p = 0.7 and 0.6, p = 0.8). CSP rate, calculated with pooled data from the three methods and species, was 3.0% in Dielmo and 4.1% in Bandafassi.

**Table 2:** Number of circumsporozoite protein (CSP) positive mosquito and corresponding CSP rate (%) according to the method (HLC: human landing catches, PSC: pyrethrum spray catches, LT/N Light trap associated with a person sleeping under a net), the village (Dielmo and Bandafassi) and the Anopheles species (*An. funestus, An. gambiae *and *An. nili*)

Species	DIELMO			BANDAFASSI		
	
	HLC	PSC	LT/N	HLC	PSC	LT/N
*An. funestus*	20 (2.96%)	11 (4.00%)	28 (2.85%)	1 (1.79%)	0 (0.00%)	2 (3.70%)
*An. gambiae*	6 (2.74%)	5 (3.05%)	5 (2.96%)	66 (4.24%)	21 (5.37%)	39 (4.09%)
*An. nili*	-	-	-	8 (3.59%)	0 (0.00%)	0 (0.00%)
**Total**	26 (2.91%)	16 (3.64%)	33 (2.86%)	75 (4.09%)	21 (4.79%)	41 (3.95%)

### Transmission

**Figure 2:**
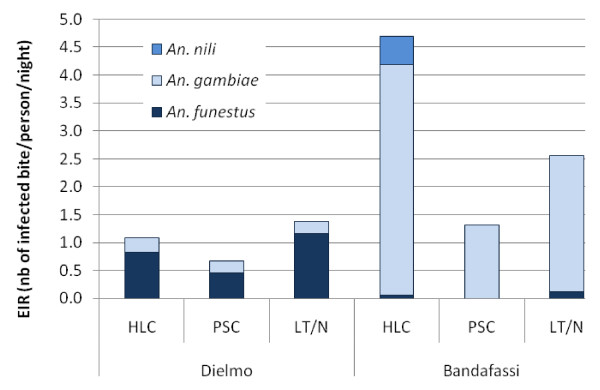
**Entomological inoculation rate (EIR, number of infected bit/person/night) according to the method (HLC: human landing catches, PSC: pyrethrum spray catches, LT/N Light trap associated with a person sleeping under a net), the village (Dielmo and Bandafassi) and the *Anopheles *species (*An. funestus, An. gambiae *and *An. nili*)**.

EIR calculated in the two villages according to the method used are represented on Figure 2. EIR measured with PSC was lower than those obtained with the other methods. EIR obtained with HLC was higher than LT/N in Bandafassi, where transmission is mainly due to *An. gambiae*. In Dielmo, where transmission is mainly due to *An. funestus*, EIR obtained by LT/N was higher.

### Correlations between the two most efficient methods

**Figure 3:**
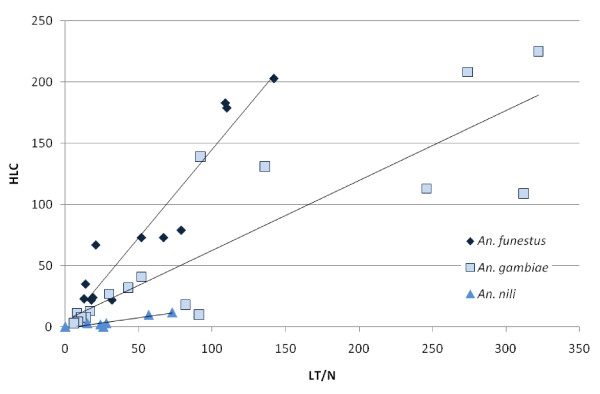
**Correlation between mean number of *Anopheles *collected by light traps assocoated with a person sleeping under a net (LT/N) and by Human Landing Catches (HLC) for the 3 species: *An. funestus, An. gambiae *and *An. nili***.

Since LT/N displayed the nearest results to HLC, the correlation between those two methods was studied (Figure 3). A significant correlation was observed for all species. The highest correlation coefficient was obtained for *An. funestus *(Spearmann rho^2 ^= 0.88, p < 0.0001). For *An. gambiae*, it was 0.74 (p < 0.0001) and for *Anopheles nili *0.64 (p = 0.02). The parameters of the linear correlation wildly differed between species, confirming performance variations of each method according to the species.

## Discussion

A large variety of traps have been developed for entomological studies, in order to avoid using human bait. According to the study, their relative efficiency, compared to HLC has been highly variable [14-18]. The aim of this study was to identify the best method to use for entomological studies in two Senegalese villages where malaria epidemiology is currently studied.

This work confirms that the method influences the quantity and the variety of mosquitoes collected. Among the three methods compared, all allowed to collect the principal known malaria vectors in studied areas: *An. gambiae s.l*. and *An. funestus *in Dielmo [8], those two species as well as *An. nili *in Bandafassi [9]. As previously reported [14], the variety of species collected with LT/N was higher than that obtained with HLC and PSC in the two villages. This is probably due to the multiple attraction stimuli displayed by the method (light, odour...). As a consequence, LT/N would be the method to be used in biodiversity studies where a complete panel of *Anopheles *species is requested.

Significant differences in *Anopheles *density were observed according to the method used, with HLC being more performing than PSC and better than or equal to LT/N. Results obtained were different according to the vector species. On the other hand, they were similar in the two villages.

For *An. gambiae s.l*., HLC was the most efficient method probably because this species is highly anthropophilic and less influenced by light attraction [9]. Whereas studies performed in areas where HBR is very low (2-6 per person and per night) demonstrated no correlation between LT and HLC [15,16], various studies, performed in areas where *Anopheles *density is higher, report that LT display results that are proportional to those obtained with HLC [17-24]. This study confirmed a good correlation between the two methods for *An. gambiae *sampling (r^2 ^= 0.74) for an HBR (measured by HLC) ranging from 18 *An. gambiae *per person and per night in Dielmo to 194 in Bandafassi. In other studies, the relative efficiency of LT using HLC as a reference was highly variable among those reporting a good correlation. It was 1.7-1.9 in two of them [17,20], 1.2 in one [21] and only 0.7 in others [18,19,24]. In this work the efficiency ratio between LT/N and HLC was 0.6. In conclusion, to sample *An. gambiae*, although HLC is the reference method, LT/N may be an alternative, only when density is sufficient.

For *An. funestus*, LT/N was the most efficient method in comparison to HLC and PSC. This is probably related to the high attraction exerted by light on this species, that is less anthropophilic than *An. gambiae *[25]. For *An. funestus*, the correlation between results obtained by LT and HLC was good in some studies [18,17,22,24] but not in another one where HBR was very low (0.04 *An. funestus *per person and per night) [15]. This study confirms a good correlation between the two methods for *An. funestus *HBR ranging from 7 bites per person and per night in Bandafassi to 56 in Dielmo. The relative efficiency of LT using HLC as a reference was highly variable among studies identifying a good correlation: 0.7 [24], 1.1 [18] 1.9 [17]. In this work it was 1.4. In conclusion, to sample *An. funestus*, LT/N seems to be a good alternative to HLC especially when density is sufficient.

In this study, the mean anopheles density was lower with PSC for all species. This collecting technique is often used in entomological studies to catch fed indoor-resting females. It cannot be considered as a quantitative method to determine aggressive anopheles density. Indeed, it tends to miss the mosquitoes that leave the house after feeding and includes those entering the house after feeding outdoor [26]. Moreover, this technique is not standardized (different insecticide may be used, time when collection is performed differs, dispersion of specimens is possible if holes are present in the walls...). In areas where an important resistance to insecticide is detected, it is possible that a part of mosquitoes present in the room will not be collected by PSC.

In this study, no influence of the method used was detected on the infection rates measured. Contradictory results have been reported concerning this parameter. In some previous studies, CSP rates were not significantly different when estimated by HLC and LT/N [17,27]. In others it was twice higher in LT than in HLC [16,21], probably because light traps tended to attract and capture resting mosquitoes that have a higher sporozoite rate than host-seeking ones [26]. In this study, since infection rates were similar among methods and species, EIR variations followed the anopheles density. In Dielmo, where *An. funestus *was the principal vector, LT/N was the method that reported the higher EIR. On the other hand, in Bandafassi where *An. gambiae *s.l. was the main vector, EIR calculated with HLC was higher.

## Conclusions

In order to have at disposal reliable entomological data, it is important to choose carefully the method used to collect mosquitoes according to the study area and, more specifically, according to vector species responsible for transmission. No method seems as reliable as HLC for measuring malaria transmission. It is possible, but difficult, to directly extrapolate the results obtained by a method to another one since coefficients vary according to species. In the studied areas, our work clearly demonstrates a good correlation between HLC and LT/N for *Anopheles *densities and CSP rates. In the future, if HLC have to be stopped, for ethical reasons, the study of malaria transmission in these areas could be performed by using light traps associated with a person sleeping under a net.

## Conflict of interest

The authors declare that they have no competing interests.

## Authors' contributions

CS and JFT equally contributed to the design and the conception of study and provided the scientific supervision. MON, AG and CB conducted field activities and molecular biology study. MON and CM analysed data and drafted the manuscript. LK and OF contributed to the analysis and interpretation of data. All authors read and approved the final manuscript.
